# MGMT autoantibodies as a potential prediction of recurrence and treatment response biomarker for glioma patients

**DOI:** 10.1002/cam4.2346

**Published:** 2019-06-17

**Authors:** Haibin Wu, Zhitong Deng, Hao Wang, Xuetao Li, Ting Sun, Zhennan Tao, Lin Yao, Yanping Jin, Xiaoying Wang, Lan Yang, Hongwei Ma, Yulun Huang, Youxin Zhou, Ziwei Du

**Affiliations:** ^1^ Department of Neurosurgery & Brain and Nerve Research Laboratory The First Affiliated Hospital of Soochow University Suzhou Jiangsu; ^2^ Nano‐Bio‐Chem Centre Suzhou Institute of Nano‐Tech and Nano‐Bionics, Chinese Academy of Sciences Suzhou Jiangsu

**Keywords:** autoantibodies, MGMT, prediction of recurrence, treatment response

## Abstract

**Background:**

Cancer‐specific autoantibodies found in serum of cancer patients have been characterized as potential predictors of the high risk of recurrence and treatment response. The objective of this study is to investigate the clinical utility of serum O‐6‐methylguanine‐DNA methyltransferase (MGMT) autoantibodies as novel biomarkers for prediction of recurrence and treatment response for glioma through MGMT peptides microarray.

**Methods:**

A total of 201 serum samples of glioma patients with various WHO grade and 311 serum samples of healthy donors were examined for the detection of MGMT autoantibodies by peptides microarray. The clinical value of MGMT autoantibodies was studied through univariable and multivariable analyses.

**Results:**

Autoantibodies to MGMT peptides were detected in sera from glioma patients and five highly responsive autoantibodies to peptides were identified in the glioma group. The positive rate of MGMT autoantibody to 20 peptides in glioma groups is compared with healthy individuals, the positive rate of MGMT‐02 (45%), MGMT‐04 (27%), MGMT‐07 (21%), MGMT‐10 (13%), and MGMT‐18 (24%) were significantly elevated in patients with glioma. MGMT autoantibody and its protein expression exhibited a significant correlation. The levels of MGMT autoantibodies decreased on the 30th day after operation, reaching preoperative levels, similar to those when tumor recurrence developed. Univariable and multivariable analyses revealed that the only preoperative autoantibodies to MGMT‐02 peptide were independently correlated with recurrence‐free survival. Preoperative seropositive patients were more likely than seronegative patients to have shorter recurrence times and to be resistant to chemoradiotherapy or chemotherapy with temozolomide.

**Conclusion:**

Monitoring the levels of preoperative serum autoantibodies to MGMT‐02 peptide was useful for predicting patients at high risk of recurrence and treatment response.

## 1 INTRODUCTION

Gliomas are the most common malignant primary brain tumors, which represent approximately 30% of all central nervous system (CNS) tumors and 80% of all malignant brain tumors. Gliobastoma is the most common malignant glioma (World Health Organization [WHO] grade IV) with an incidence rate of 3.2 per 100 000 population.^1^, ^2^

O‐6‐methylguanine‐DNA methyltransferase (MGMT) is a DNA repair protein, which removes the cytotoxic O6‐methylguanine (O6‐MG) DNA lesions generated by temozolomide (TMZ), an oral methylating agent used in the treatment of primary CNS tumors and melanoma. A high MGMT expression in cells is the predominant mechanism underlying tumor resistance to alkylating agents.^3^, ^4^, ^5^, ^6^ Currently, it has been shown that MGMT methylation or protein expression can be used as useful predictive biomarkers during temozolomide chemotherapy.^7^, ^8^, ^9^, ^10^ MGMT methylation status testing by methylation‐specific PCR, immunohistochemistry (IHC) or pyrosequencing has been established as a routine molecular pathological technique for patients with glioma. Yet, these methods have several limitations, including a high false‐positive rate, difficulty in gaining access to human samples and substantial costs.^11^, ^12^ Therefore, there is an urgent need for more effective, noninvasive method for the screening of MGMT.

Autoantibodies against tumor‐associated antigens (TAAs) are attractive targets for the development of noninvasive serological tests, which have shown to be useful for predicting high risk of recurrence and/or treatment response.^13^, ^14^, ^15^, ^16^, ^17^ In the present study, we conducted a peptide microarray to examine whether humoral immunity participated in the immune process to elicit autoantibodies against MGMT in gliomas, as well as to investigate whether MGMT autoantibodies could be used as biomarkers for monitoring the recurrence and prediction of treatment response of glioma.

## 2 MATERIALS AND METHODS

### 2.1 Collection of serum samples

Serum samples were collected between January 2012 and November 2016, from patients with different WHO grade gliomas and recurrence gliomas admitted at the First Affiliated Hospital of Soochow University (Suzhou, China). The healthy control group included donors who attended routine health exams at the same hospital; these patients had no evidence of any current or prior malignant disease. Peripheral blood samples were centrifuged at 1500 rpm for 5 minutes and all samples were stored at −80°C until further analysis. The patients’ characteristics are shown in Table 1.

**Table 1:** Patient details and clinicopathological characteristics

Group	Glioma			Healthy
	Preoperative	Postoperative 30 days	Recurrence glioma	
Number, n	67	52	11	311
Gender, n (%)				
Male	37 (55.22%)	31 (59.62%)	9 (81.82%)	169 (54.36%)
Female	30 (44.78%)	21 (40.38%)	2 (18.18%)	142 (45.65%)
Mean age ± SD, years	52.07 ± 9.74	50.59 ± 12.82	47.73 ± 10.33	41.80 ± 16.03
Age range, years	19‐80	22‐75	29‐62	17‐66
WHO grades, n (%)				
II	16 (23.89%)	11 (21.15%)	3 (27.27%)	—
III	33 (49.25%)	22 (42.31%)	6 (54.55%)	—
IV	18 (26.86%)	19 (36.54%)	2 (18.18%)	—

The Ethics Committee of the First Affiliated Hospital of Soochow University approved this work. Recurrent gliomas were diagnosed by Magnetic Resonance Imaging.^18^, ^19^, ^20^

### 2.2 MGMT peptides library

MGMT amino acid sequence information was obtained from the NCBI website (Sequence ID: AAP36645.1). The peptides overlap by 10‐amino acid with 10‐amino acid offset. MGMT peptides and corresponding amino acids sequences are shown in Table 2.

**Table 2:** MGMT peptides and their corresponding amino acids sequences

Peptides	Start position	End position	Sequence
MGMT‐01	1	20	MDKDCEMKRTTLDSPLGKLE
MGMY‐02	11	30	TLDSPLGKLELSGCEQGLHE
MGMT‐03	21	40	LSGCEQGLHEIKLLGKGTSA
MGMT‐04	31	50	IKLLGKGTSAADAVEVPAPA
MGMT‐05	41	59	ADAVEVPAPAAVLGGPELM
MGMT‐06	51	68	AVLGGPELMQCTAWLNAYF
MGMT‐07	60	79	QCTAWLNAYFHQPEAIEEFP
MGMT‐08	70	89	HQPEAIEEFPVPALHHPVFQ
MGMT‐09	80	99	VPALHHPVFQQESFTRQVLW
MGMT‐10	90	109	QESFTRQVLWKLLKVVKFGE
MGMT‐11	100	119	KLLKVVKFGEVISYQQLAAL
MGMT‐12	110	129	VISYQQLAALAGNPKAARAV
MGMT‐13	120	139	AGNPKAARAVGGAMRGNPVP
MGMT‐14	130	149	GGAMRGNPVPILIPCHRVVC
MGMT‐15	140	159	ILIPCHRVVCSSGAVGNYSG
MGMT‐16	150	169	SSGAVGNYSGGLAVKEWLLA
MGMT‐17	160	179	GLAVKEWLLAHEGHRLGKPG
MGMT‐18	170	189	HEGHRLGKPGLGGSSGLAGA
MGMT‐19	180	199	LGGSSGLAGAWLKGAGATSG
MGMT‐20	190	206	WLKGAGATSGSPPAGRN

### 2.3 Reagents

15 × 15 mm^2^ polymer coated initiator integrated poly (dimethysiloxane) membrane (iPDMS) was purchased from Epitope‐Bio (Suzhou, China). 1‐Ethyl‐3‐(3‐(dimethylamino) propy 1) carbodiimide (EDC) and N‐hydroxysuccinimide were obtained from Medpep (Shanghai, China). MGMT peptides (20mers) were chemically synthesized by GL Biochem (Shanghai, China). Human IgG (H‐IgG) was purchased from DGCS‐Bio (Beijing, China). Horseradish peroxidase‐labeled goat antihuman IgG (HRP‐IgG) was obtained from ZSGB‐Bio (Beijing, China). Peroxidase conjugate stabilizer/diluent and chemiluminescence substrates (SuperSignal ELISA Femto Maximum Sensitivity Substrate) were purchased from Thermo Fisher Scientific (Pierce Protein Biology Products, San Diego, CA, USA).

### 2.4 Peptide microarray

**Figure 1:**
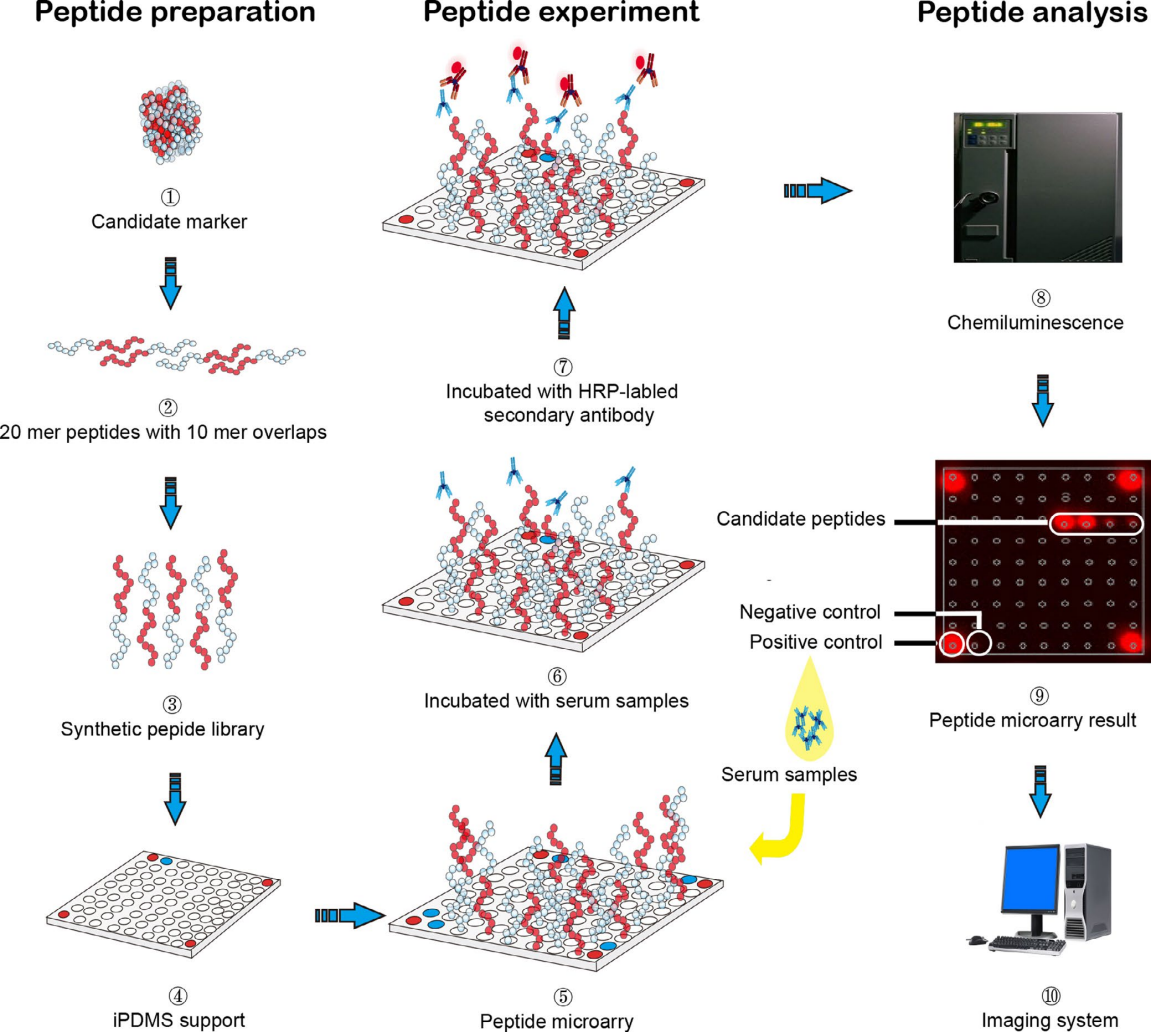
Flow chart of serological screening of MGMT peptides. Preparation of MGMT peptide microarray were divided in four steps (1‐4): (1) MGMT proteins were selected; (2) proteins were cut into peptides that were 20 mers with 10aa overlap peptides (blue and red); (3) MGMT peptides were chemically synthesized; (4) MGMT peptides were designed for the microarray. All spots were organized as a 9 × 9 array with peptides probes (blank circle), positive controls (red circle), and negative controls (blue circle). (4) Printed on iPDMS membrane to form microarrays. MGMT peptide microarray experiment (6‐7): (6) Incubation with serum samples; (7) HRP‐labeled secondary antibody. MGMT peptide microarray analysis by chemiluminescence (8‐10): (8) Antibody binding to target peptide was acquired and processed to chemiluminescence immunoassay picture by CCD camera; (9) Chemiluminescence immunoassay picture shown is the result of one of the glioma patients; (10) Further analysis by computer

Preparation of peptides microarray and serum screening were according to previously described approach.^21^, ^22^ Briefly, synthetic peptides were spotted onto the activated iPDMS membranes to form a 9 × 9 microarrays using a contact printer Smart 48 (Capitalbio, Beijing, China). Human‐IgG (H‐IgG, DGCS‐Bio, Beijing, China) was used as positive control in each subarray at the concentration of 50 μg/mL, while printing buffer served as negative control. In each subarray there were four positive controls printed with H‐IgG at the concentration of 50 μg/mL, and one negative control printed with printing buffer (Figure 1 and Table S1).

### 2.5 Serological screening

A total of 2‐μL serum samples were diluted 1:100 with a sample dilution buffer and incubated at 200 μL per well for 2 hours with shaking (150 rpm, 22°C) with no blocking as previously reported.^21^ The microarray was then rinsed 3 times with TBST (20‐mmol/L Tris‐HCl, pH 6.8, 137‐mmol/L NaCl, 0.1% Tween 20) and incubated with 200‐μL horseradish peroxidase HRP‐labeled goat antihuman IgG (ZSGB‐Bio, Beijing, China) at a 1:25 000 dilution with peroxidase conjugate stabilizer/diluent (Thermo Fisher, Waltham, MA) for another 1 hours. Consequently, Super Signal ELISA Femto Maximum Sensitivity Substrate (Thermo Fisher, Cat.no.37075) (15 μL) was added to the microarray; then chemiluminescence signals were acquired at a wavelength of 635 nm using a LAS4000 imaging system (GE Healthcare, Waukesha, WI). Signals were finally saved as images in TIFF format. Five peptides that were most likely to be targeted by autoantibodies were selected.

### 2.6 Immunohistochemistry (IHC) for MGMT and Ki‐67 protein

A total of 21 tissues samples of glioma patients (WHO grade II, n = 3; WHO grade III, n = 4; WHO grade IV, n = 14) were fixed in 4% buffered formaldehyde for 24 hours at 4°C. Paraffin‐embedded tissue sections (5‐μm thickness) were placed on positively charged slides and air‐dried. Slides (sections) were deparaffinized at 60°C followed by xylene changes. Endogenous peroxidase activity was blocked with 0.3% H_2_O_2_ in methanol for 30 minutes. Slides were incubated at 4°C overnight with a mouse antibody against human primary antibody. The slides were incubated with primary antibodies, including Ki‐67 (1:50; Santa cruz, Biotechnology, CA) and MGMT (1:250; Santa cruz, Biotechnology, CA). Diaminobenzidine was used to catch the signal followed by a nuclear stain (hematoxylin: blue). Assessment and scoring of MGMT expression in tumor sections was method based on percent of cell nuclei that were positive: negative (≤10% of cells positively stained), positive (>10% of cells positively stained). For the results of Ki‐67 expressions through the method of IHC, we classified it into low (≤20% of cells positively stained) and high (>20% of cells positively stained) Ki‐67 expressions by evaluating the staining percentage of tumor cells. Immunohistochemistry (IHC) was conducted by pathologists in the department of pathology. The examining results were verified by at least two pathology experts individually.

### 2.7 Statistical analysis

Each microarray image was processed using GenePix Pro 6.0 software to calculate the median chemiluminescence intensity of each dot, which was converted to signal‐to‐noise ratio (SNR) by subtracting the background intensity averaged from the intensity from eight blank dots, and calculated using the following formula: *(signal intensity‐background intensity)/(background intensity)*. The digitized image from each microarray was then imported into R for further analysis. The R package “p‐heatmap” was used for cluster analysis. The cut‐off values of each peptide autoantibodies were determined as the SNR that yields maximum difference of positive response rate between glioma and healthy normal group. The differences between groups were analyzed by Fisher's exact test; Bonferroni corrections were performed for multiple comparisons. Samples with SNR ≥ each peptide cut‐off value were considered as seropositive reaction. The coverage of a peptide to a set of serum samples was defined as the ratio of the seropositive serum numbers to the whole set numbers. Correlation among variables was assessed using the nonparametric Spearman coefficient. A *P* < 0.05 was considered statistically significant.

Recurrence‐free survival (RFS) was defined as the interval from the date of surgery to the date of glioma recurrence as a time‐to‐event end point. Survival curves were estimated by the Kaplan‐Meier method and compared using the log‐rank test. Hazard ratios with 95% confidence intervals were calculated after univariate and multivariate analysis using the multiple Cox regression analysis with forward selection. All statistical tests were two‐sided, and *P* < 0.05 indicated a significant difference. These analyses were performed using the statistical software with SPSS (version 21, SPSS Company, Chicago, IL).

## 3 RESULTS

### 3.1 Primary analysis of candidate sequences

**Figure 2:**
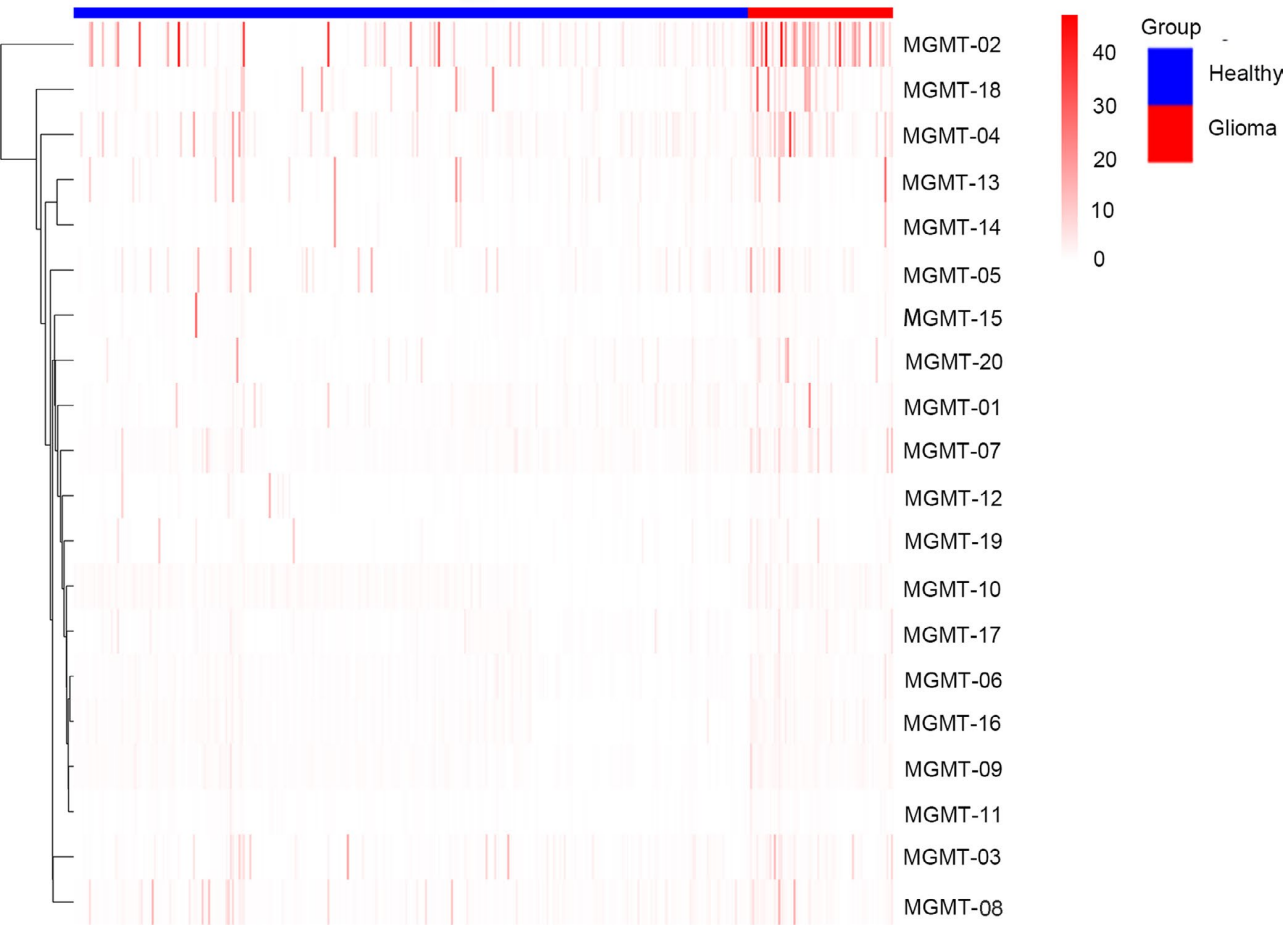
Cluster analysis of the normalized IgG responses. Normalization operation of the SNR value of each peptide was carried out in order to adjust the data for systematic errors that arose from experimental and not biological variation. Normalized IgG responses of glioma and healthy individuals to 20 MGMT peptides were clustered, respectively. Only one recognition pattern emerged peptides having significantly higher response in glioma individuals. Heatmap responses from 378 serum samples (67 glioma patients and 311 healthy control samples) to the identified 5 peptides. The responses were obtained and clustered by SNR. We could easily identify the differences between the two groups in the serological screening of MGMT peptides from the heatmap

The peptides microarrays were incubated with the sera from 378 subjects (67 glioma patients and 311 healthy donors) were used for the serological screening of MGMT autoantibody peptides. The bound IgG was detected by HRP‐conjugated secondary antibody. Each serum sample was incubated with two peptide microarrays. The results of microarray screening between glioma group and healthy group are shown in Figure S1. Cluster analysis of the two groups was carried out, revealing that peptides showed significantly higher responses in glioma than healthy individuals (Figure 2). The cut‐off value of each peptide autoantibody and maximum difference of positive response rate between glioma and healthy normal group are shown in Table 3.

**Table 3:** Difference of positive response rate between two groups

Peptides	Cut‐off value	Difference^a^	Response (ration)		*P*‐value
			Glioma (n = 67)	Healthy (n = 311)	
MGMT‐01	2.4	10%	8 (12%)	7 (2%)	0.0015
MGMT‐02	5.5	39%	30 (45%)	19 (6%)	9.29 × 10^−14^
MGMT‐03	2.7	11%	10 (15%)	11 (4%)	0.001122
MGMT‐04	3.1	22%	18 (27%)	16 (5%)	8.48 × 10^−07^
MGMT‐05	2.0	7%	9 (13%)	20 (6%)	0.072271
MGMT‐06	3.0	4%	3 (4%)	0 (0)	0.005364
MGMT‐07	2.2	19%	14 (21%)	6 (2%)	1.44 × 10^−07^
MGMT‐08	2.0	6%	9 (13%)	22 (7%)	0.090275
MGMT‐09	2.0	3%	3 (4%)	2 (1%)	0.040836
MGMT‐10	2.0	12%	9 (13%)	1 (1%)	9.31 × 10^−07^
MGMT‐11	2.0	0	1 (1%)	1 (1%)	0.323467
MGMT‐12	3.0	0	1 (1%)	3 (1%)	0.543355
MGMT‐13	5.5	4%	4 (6%)	6 (2%)	0.08156
MGMT‐14	2.2	2%	2 (3%)	3 (1%)	0.216133
MGMT‐15	2.0	0	1 (1%)	1 (1%)	0.323467
MGMT‐16	2.0	8%	6 (9%)	3 (1%)	0.001368
MGMT‐17	3.4	3%	3 (4%)	2 (1%)	0.040836
MGMT‐18	2.0	19%	16 (24%)	15 (5)	6.43 × 10^−06^
MGMT‐19	2.0	6%	5 (7%)	4 (1%)	0.010728
MGMT‐20	2.7	8%	7 (10%)	7 (2%)	0.004965

a Different = positive response rate of glioma group－positive response rate of healthy normal group.

### 3.2 Correlation of MGMT autoantibody and its Protein Expression

MGMT autoantibody and its protein expression were performed on same series of glioma samples. Samples with SNR ≥ each peptide (MGMT‐02, MGMT‐04, MGMT‐07, MGMT‐10, MGMT‐18) cut‐off value were considered as seropositive reaction. The results showed significant positive correlation between MGMT autoantibody seropositive reaction and its protein expression with a Spearman Correlation Coefficient of *r* = 0.553 and *P* < 0.001 values (Table 4).

**Table 4:** Association of MGMT MGMT protein expression and MGMT autoantibody seropositive reaction of Glioma patients

Variables		MGMT expression		Spearman correlation coefficient	
		Expression	No expression	*r*	*P* value
MGMT autoantibody	Seropositive	4	3	0.553	<0.001
	Seronegative	1	13		

### 3.3 The changing of preoperative, postoperative, and recurrence MGMT autoantibodies

**Figure 3:**
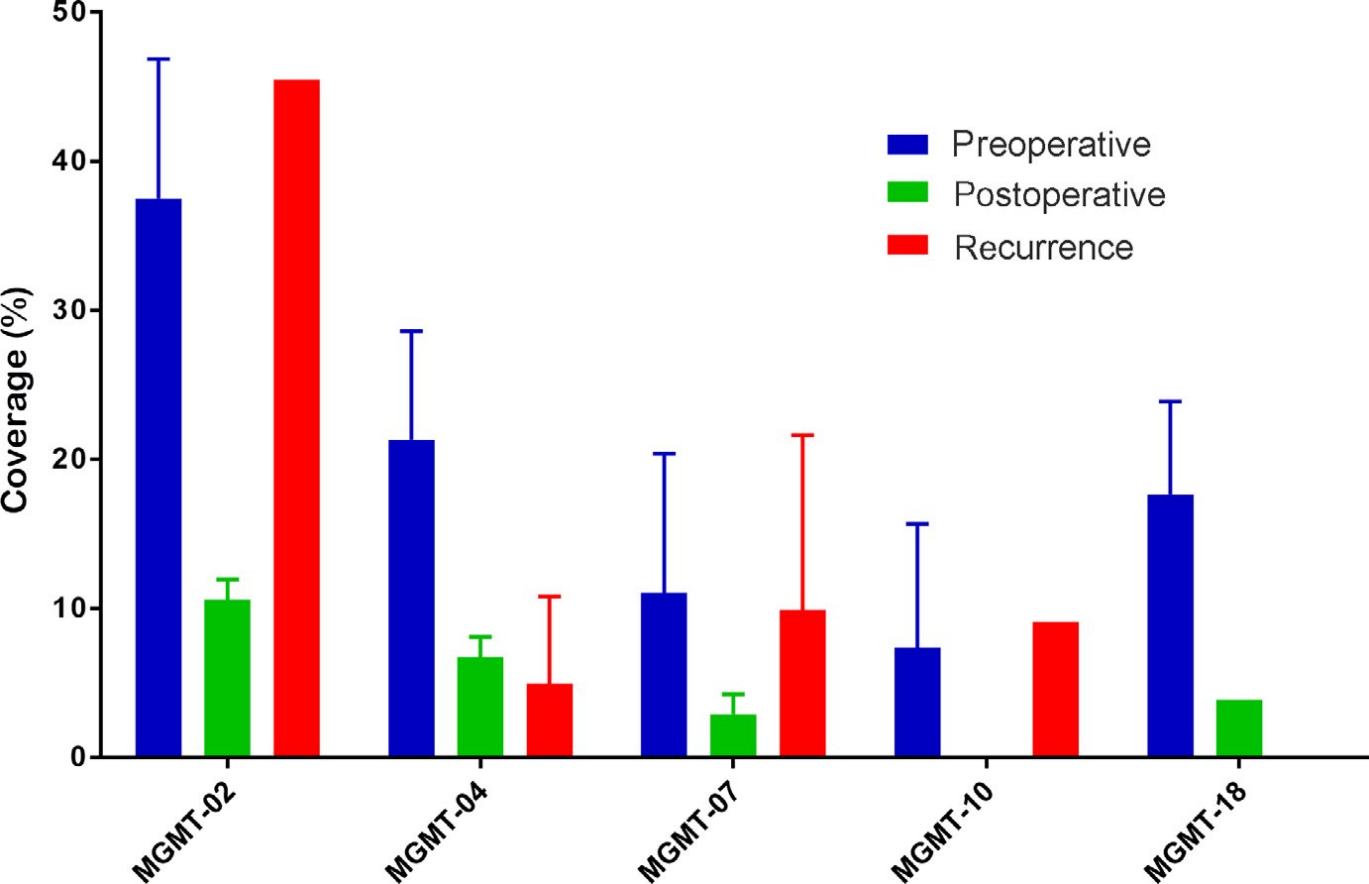
Autoantibodies to MGMT peptides response to glioma in different time points (preoperative, postoperative and recurrence). The coverage ratio of autoantibodies to MGMT peptide response to the sera collected at different times

**Figure 4:**
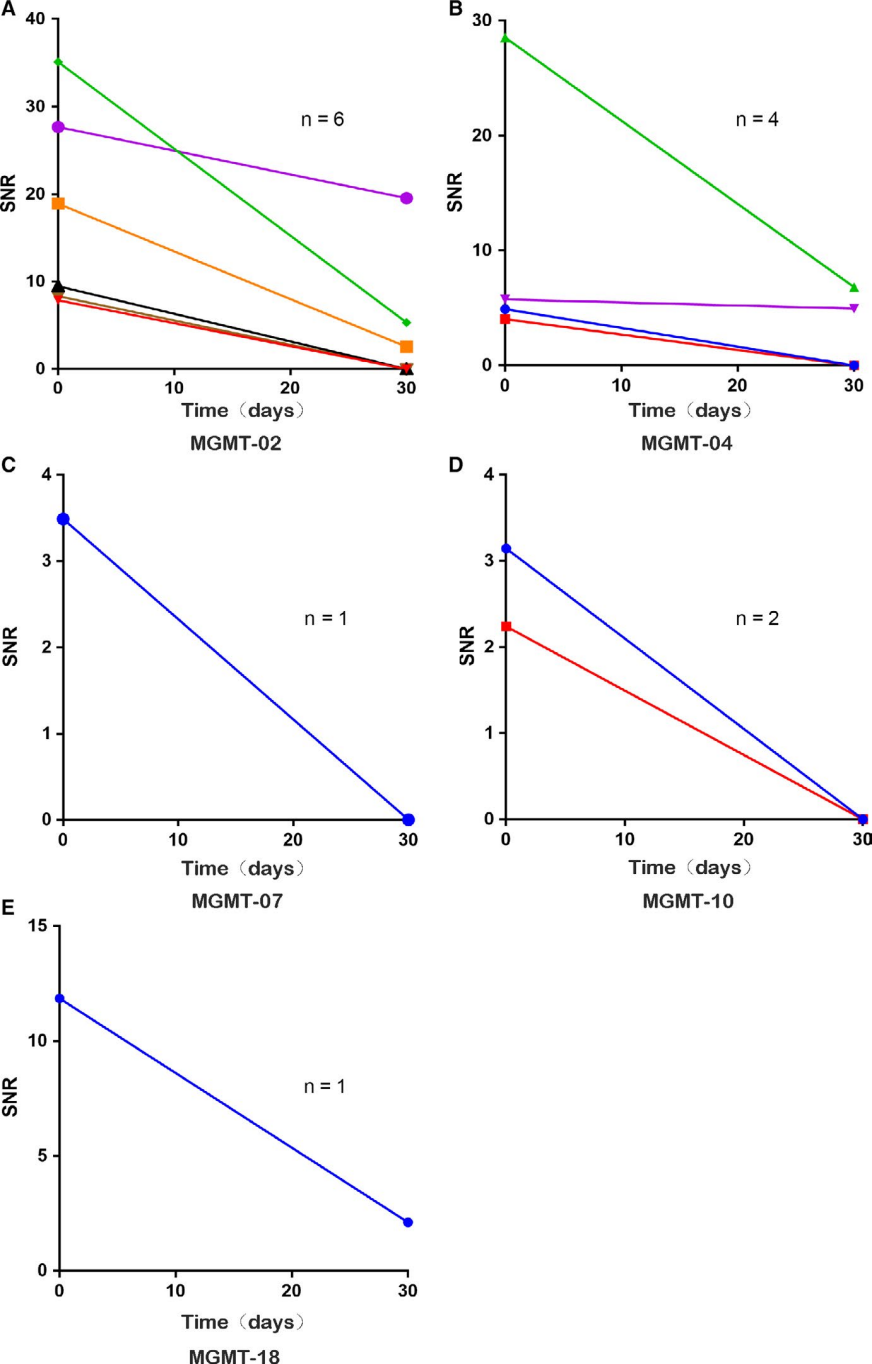
The changing of SNR values in the autoantibodies to 5 peptides to the sera collected before and 30 days after operation in 10 glioma patients. Autoantibodies to MGMT‐02 (A, n = 6), MGMT‐04 (B, n = 4), MGMT‐07 (C, n = 1), MGMT‐10 (D, n = 2), MGMT‐18 (E, n = 1)

We used the collected 52 serum samples of glioma 30 days postoperatively, and 11 serum samples of recurrent gliomas detected the five‐peptide responses necessary for investigation of the changing regularity of serum MGMT autoantibodies, which are shown in Figure 3. We observed that the autoantibody coverage of peptide in five peptides was lower at 30 days postoperatively than preoperatively. In 10 patients whose MGMT autoantibodies were positive preoperatively, the sera autoantibody levels were also examined 30 days after surgery revealing that decreased levels of five peptide autoantibodies (Figure 4).

**Figure 5:**
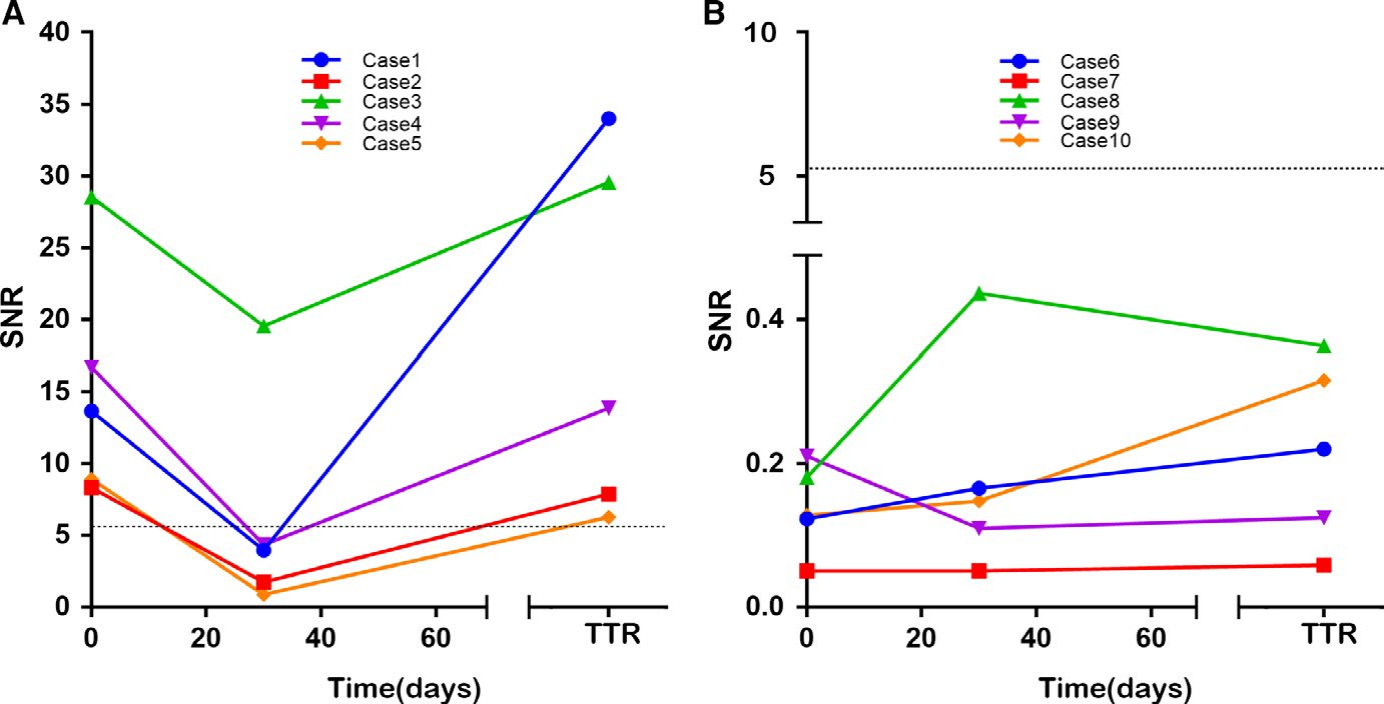
The changing of SNR values in the autoantibodies status to MGMT‐02 peptide before and after operation, and recurrence in 10 glioma patients. Seropositive patients (A, n = 5), seronegetive patients (B, n = 5). The dotted line indicates the cut‐off value. Abbreviations: TTR, Time to Recurrence

When the tumor reoccurs, we found that the anti‐MGMT‐02, anti‐MGMT‐07, and anti‐MGMT‐10 peptide autoantibodies coverage of peptide increased. However, only anti‐MGMT‐02 peptide autoantibodies had a higher coverage of peptide than the preoperative when tumor recurrence developed. We also followed up 10 glioma patients sera (5 seropositive patients and 5 seronegative patients of preoperative) autoantibody levels during postoperative 30 days and the tumor recurrence to validate the changing of anti‐MGMT‐02 peptide autoantibody level (Figure 5). Among 5 seropositive patients, the anti‐MGMT‐02 autoantibody peptide level decreased 30 days after surgery; 4 out of the 5 seropositive patients became seronegative, 5 patients with anti‐MGMT‐02 peptide autoantibodies reached preoperative levels again when tumor recurrence developed (Figure 5A). In 5 seronegative patients, anti‐MGMT‐02 peptide autoantibody level remained seronegative; not only 30 days postoperatively but also when tumor recurred (Figure 5B).

Monitoring of anti‐MGMT‐02 peptide autoantibody levels was useful for identifying patients with glioma recurrence from preoperative seropositive patients.

### 3.4 MGMT autoantibody status and level in association with Treatment Response in entire glioma population

**Figure 6:**
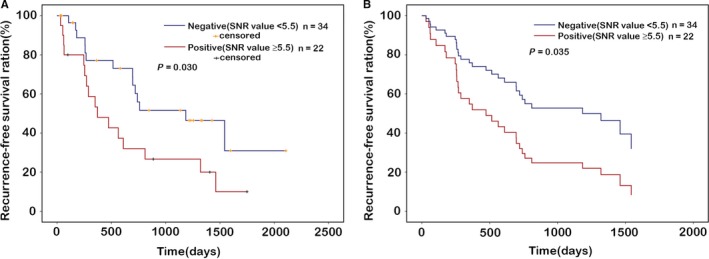
Recurrence‐free survival curves of patients according to autoantibodies to MGMT‐02 peptide levels after combined radio‐chemotherapy with temozolomide. Negative group (SNR value <5.5), positive group (SNR value ≥5.5). In the univariate analysis (A) and multivariate analysis (B), MGMT‐02 peptide was significantly correlated to the recurrence‐free survival of patients. *P* < 0.05 was considered statistically significant

We evaluated the clinical impact of MGMT autoantibody status and the level of the prediction of recurrence‐free survival (RFS) in 56 glioma patients with various grade (WHO grade II, n = 16; WHO grade III, n = 25; WHO grade IV, n = 15). All glioma patients received operative intervention (mean resection rate was 95%), and chemoradiotherapy and chemotherapy with temozolomide according to the NCCN regimen.^23^, ^24^, ^25^ The major factors such as sex, age, Ki‐67, and glioma grade were investigated（low‐grade glioma (WHO grade II) and high‐grade glioma (WHO grade III‐ IV). The status of MGMT peptide autoantibodies was divided into two groups according to the cut‐off value of each peptide, negative group (SNR value < cut‐off value) and positive group (SNR value > cut‐off value). Univariate and multivariate analysis showed that preoperative anti‐MGMT‐02 peptide autoantibodies status was an independent risk factor for RFS; the negative group showed significantly better recurrence‐free survival than the positive group (*P* < 0.05; Figure 6A,B), suggesting the possibility of using the preoperative anti‐MGMT‐02 peptide autoantibodies as a predictive marker of recurrence. Patient’ age, gender, Ki‐67, and glioma grade were not significant variables (Table 5).

**Table 5:** Multivariable survival analysis for RFS

Variables	HR	*P*‐value	95% CI
Age, <65 or ≥65	0.846	0.835	0.175‐5.078
Gender	1.093	0.831	0.488‐2.442
WHO Glioma Grade^a^	1.854	0.288	0.549‐5.791
Ki‐67, <20% vs. ≥20%	1.260	0.613	0.514‐3.088

Abbreviations: CI, confidence interval; RFS, recurrence‐free survival; HR, hazard ratio.

a WHO Glioma Grade: Lower Grade Glioma vs. High Grade Glioma.

Perioperative monitoring of anti‐MGMT‐02 peptide autoantibody levels was useful for identifying patients with glioma at poor prognosis and a high risk of tumor recurrence and poor prognosis.

## 4 DISCUSSION

Monitoring treatment response and recurrence, prompt treatment regimen can be used to reduce mortality and improve prognosis. Unfortunately, this can be challenging because of the low specificity and sensitivity of the less invasive methodologies currently available, and because refined diagnosis requires resection or biopsy to obtain tumor tissue for genetic or IHC biomarkers. Yet, repeated sampling of tumor tissue is not always appropriate because it is an invasive approach, which affects the clinical diagnosis and design of individualized treatment ultimately compromising the treatment effect.^26^

Our results showed that the autoantibodies to MGMT peptides can be detectable. Furthermore, we identified five highly responsive peptides in the glioma sera. The mechanism underlying anti‐MGMT peptides autoantibody production still remains unclear; however, it might be associated with the MGMT protein overexpression. Because tumor development is often accompanied with the overexpression of tumor‐associated antigens (TAAs) which may elicit immune responses resulting in the production of anti‐TAA autoantibodies, even when antigen expression is minimal, serum autoantibodies can be detected.^27^ Our present study also showed MGMT autoantibody and its protein expression exhibited a significant correlation (Spearman's *r* = 0.553, *P* < 0.001).

We validated its value in patients at different time points learning that the coverage ratio of all five autoantibody peptides had decreased 30 days after operation compared to levels before surgery; however, only MGMT‐02 peptide reached preoperative levels when tumor recurrence developed. When the tumor was resected, the antigen in the body was removed, causing the immune response to be reduced, so the expression of peptides was decreased. Therefore, these cancer‐associated autoantibodies might be considered as reporters from the immune system, that can identify the antigenic changes of cellular proteins involved in the transformation process.^28^, ^29^ When the glioma recurred, the tumor cells secreted antigens, and the peptides increased. In our study, we also found that peptides were increased before recurrence. Therefore, monitoring anti‐MGMT‐02 peptides autoantibody could be valuable for the recurrence of glioma.

We also investigated the relationships between preoperative MGMT autoantibody and treatment response in high‐grade glioma patients who underwent surgery following radiochemotherapy with temozolomide. We found that the seropositivity of MGMT‐02 peptide autoantibody in glioma patient serum was associated with shorter recurrence‐free survival compared with the seronegative patients. The preoperative anti‐MGMT‐02 peptide autoantibody levels were significantly correlated with the effect of the postoperative for chemotherapy with temozolomide, further indicating its clinical utility for monitoring the response to therapy.

There are several limitations in the present study. First, it is a retrospective study conducted at one single institute, where only 67 glioma patients were included in our filter set, which might cause the limitation of our findings. Second, a large multicenter study investigating the validity of the cut‐off value of each peptide autoantibody, the association between the preoperative MGMT autoantibody level and RFS would enhance the findings of the current study and provide crucial information on the utility of MGMT autoantibody levels as a prognostic marker for glioma recurrence and treatment response.

## CONFLICT OF INTEREST STATEMENT

No potential conflict of interest was disclosed.
