# Supplementary figures and images for: MGMT autoantibodies as a potential prediction of recurrence and treatment response biomarker for glioma patients
Source: Cancer Med. 2019 Jun 17;8(9):4359–69. doi: 10.1002/cam4.2346 (PMC6675704; doi:10.1002/cam4.2346)

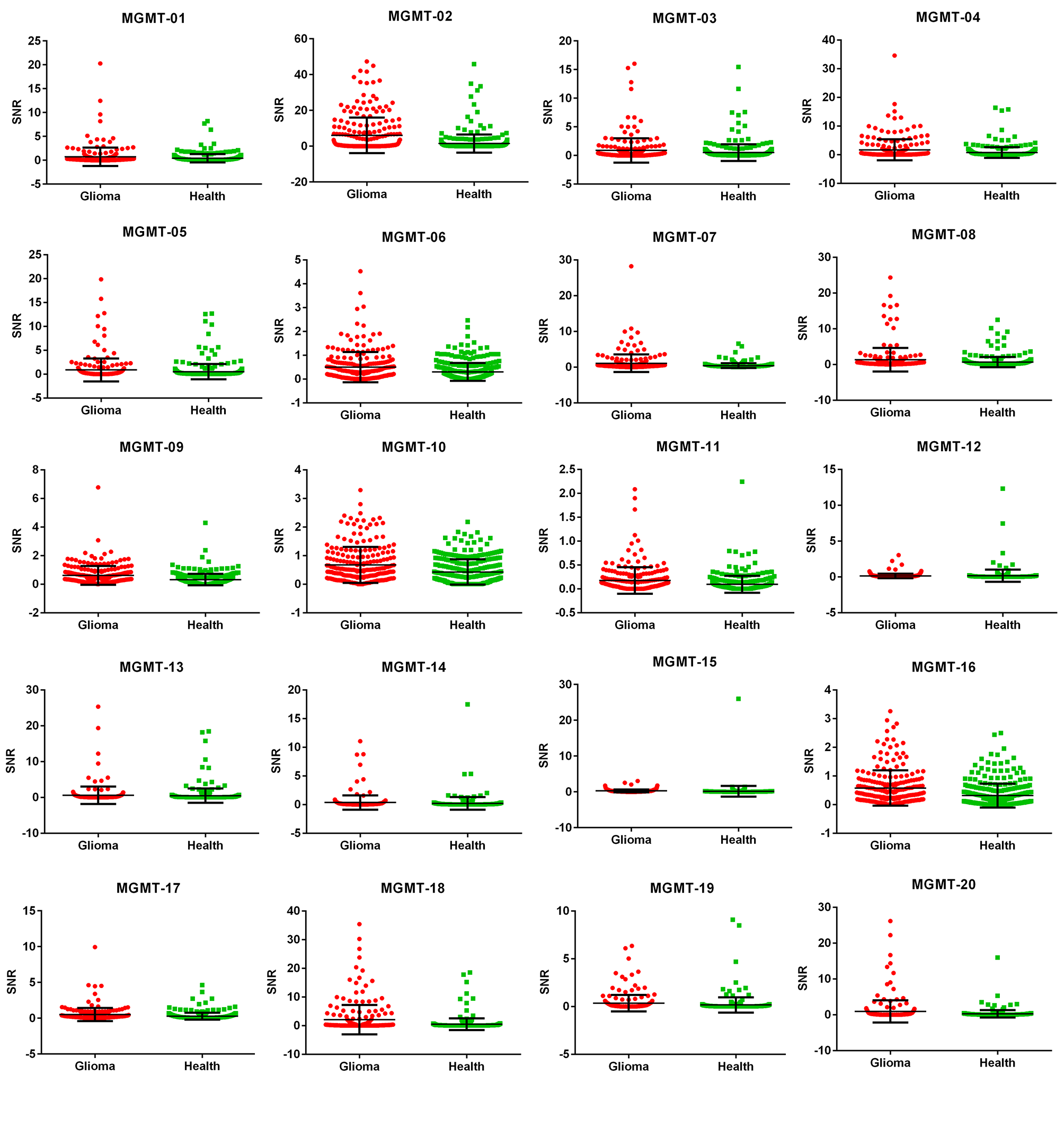

Supplement: Supplementary file 1 [file CAM4-8-4359-s001.tif]
