# Supplementary material for: MGMT autoantibodies as a potential prediction of recurrence and treatment response biomarker for glioma patients
Source: Cancer Med. 2019 Jun 17;8(9):4359–69. doi: 10.1002/cam4.2346 (PMC6675704; doi:10.1002/cam4.2346)
Supplement: Supplementary file 2 [file CAM4-8-4359-s002.docx]

**Supplementary Table 1. The layout of MGMT peptide in the subarray.**

|  | X1 | X2 | X3 | X4 | X5 | X6 | X7 | X8 | X9 |
| --- | --- | --- | --- | --- | --- | --- | --- | --- | --- |
| Y1 | Positive control | Negative control |  |  |  |  |  | Negative control | Positive control |
| Y2 |  |  | MGMT-01 | MGMT-02 | MGMT-03 | MGMT-04 | MGMT-05 |  |  |
| Y3 | MGMT-06 | MGMT-07 | MGMT-08 | MGMT-09 | MGMT-10 | MGMT-11 | MGMT-12 | MGMT-13 | MGMT-14 |
| Y4 | MGMT-15 | MGMT-16 | MGMT-17 | MGMT-18 | MGMT-19 | MGMT-20 |  |  |  |
| Y5 |  |  |  |  |  |  |  |  |  |
| Y6 |  |  |  |  |  |  |  |  |  |
| Y7 |  |  |  |  |  |  |  |  |  |
| Y8 |  |  |  |  |  |  |  |  |  |
| Y9 | Positive control | Negative control |  |  |  |  |  | Negative control | Positive control |
